# Prevalence, Common Types, and Risk Factors of Musculoskeletal Disorders Among Female University Student Athletes: A Cross-Sectional Study

**DOI:** 10.3390/jcm14217750

**Published:** 2025-10-31

**Authors:** Sarah Bajuaifer, Muniera Alsalem, Dana Alotaibi, Shadan Alshehri, Maryam Amin, Reem M. Alwhaibi

**Affiliations:** Department of Rehabilitation Sciences, College of Health and Rehabilitation Sciences, Princess Nourah Bint Abdulrahman University, P.O. Box 84428, Riyadh 11671, Saudi Arabia; ssbajuaifer@pnu.edu.sa (S.B.); meme.2013.md@gmail.com (M.A.); dana22a22s@gmail.com (D.A.); shadanalshehri@icloud.com (S.A.); maryamsaleh000@gmail.com (M.A.)

**Keywords:** musculoskeletal disorders, female athletes, risk factors, warm-up, training intensity, injury history

## Abstract

**Background**: Musculoskeletal disorders (MSDs) are a prevalent health concern among athletes, particularly female university students, who may face sport-specific, physiological, and biomechanical risk factors. In Saudi Arabia, the increasing participation of women in organized sports underscores the need to examine the burden of MSDs in this under-researched population. **Objectives**: This study aimed to (1) determine the prevalence of MSDs among female university student athletes, (2) identify the most commonly affected body regions, and (3) investigate associations between MSD occurrence and risk factors including sport type, warm-up practices, weekly training frequency, and history of previous injuries. **Methods**: A cross-sectional study was conducted among 407 physically active female university students aged 16–25 years from multiple Saudi universities. Data were collected using the validated Arabic version of the Nordic Musculoskeletal Questionnaire (NMQ) and a structured risk factor survey. Descriptive statistics, chi-square tests, and logistic regression analyses were used to examine prevalence and associated factors. **Results**: Among 407 participants, 65.8% reported at least one MSD in the past 12 months. The most commonly affected regions were the shoulders (43.2%), neck (41.8%), and lower back (32.2%). However, multivariate logistic regression revealed that previous injury history (OR = 2.44, 95% CI: 1.47–4.06, *p* = 0.001) and higher weekly training frequency (OR = 1.23, 95% CI: 1.02–1.49, *p* = 0.034) were significant independent predictors of MSD occurrence, while participation in team sports showed a borderline association (OR = 1.59, 95% CI: 0.95–2.67, *p* = 0.079). **Conclusions**: MSDs are highly prevalent among female university student athletes in Saudi Arabia, particularly affecting the shoulders, neck, and lower back. A history of previous injury and higher weekly training frequency are identified as significant independent predictors of MSD occurrence, while team sport participation showed a borderline association that warrants further exploration. These findings highlight the need for targeted prevention strategies that emphasize rehabilitation continuity—particularly for athletes with prior injuries—and training load management for those with higher weekly training frequency to reduce the risk of MSDs and promote long-term musculoskeletal health.

## 1. Introduction

Sports participation offers young adults a variety of physical, mental, and social benefits, promoting a healthy lifestyle, especially among university students [1,2,3]. However, participation in sports also carries an increased risk of sports-related injuries [4]. Evidence suggests that female athletes may be at elevated risk for certain injury patterns due to interacting biomechanical, neuromuscular, and hormonal factors, contributing to a higher burden of musculoskeletal complaints in some settings [5,6,7].

Understanding the prevalence and risk factors of musculoskeletal disorders (MSDs) in this demographic is therefore important for prevention and care. Ivković et al. (2007) highlight the role of biomechanical factors, such as joint alignment and movement patterns, in increasing the risk of MSDs [8]. Similarly, Chidi-Ogbolu et al. (2019) emphasizes hormonal influences, including the effects of estrogen on ligament laxity, as a significant contributor to injury susceptibility [9]. Lephart (2018) further observed that estrogen levels in women peak during their mid to late 20 s, decline by 50% by the age of 50, and drop dramatically after menopause, illustrating how hormonal changes across different life stages can impact injury risk [10].

Barber-Westin & Noyes (2011) discuss the heightened vulnerability of female athletes to sports-related injuries, such as ACL injuries, attributing this to hormonal and anatomical factors that make them particularly prone to MSDs [11]. For instance, Wojtys et al. (2002) note that female athletes typically exhibit higher estrogen levels, distinct body mass compositions, and unique biomechanical movement patterns, all of which elevate their risk of MSDs [12]. Additionally, Barber-Westin & Noyes (2011) emphasize environmental factors such as training habits, sport-specific demands, and nutritional intake as contributors to MSD risk, which may vary based on the type of sport, training intensity, and warm-up practices [11,13]. Together, these findings highlight the importance of implementing targeted preventive strategies to reduce MSD risk in this population.

A survey of female high school athletes revealed a 63.1% prevalence of MSDs, with even higher rates observed in aesthetic sports such as gymnastics [14]. Among Norwegian female biathlon athletes, the prevalence of MSDs reached 57.8%, with knee injuries accounting for 23% of cases [14]. Similarly, elite female basketball players reported an MSD prevalence of approximately 50%, with frequent injuries involving lower back pain and ankle sprains [15]. These findings highlight the widespread occurrence of MSDs among female athletes and underscore the necessity for sport-specific injury prevention strategies, particularly given the elevated injury risk associated with high training loads and repetitive movements.

Modifiable factors, including warm-up practices, training intensity/load, and injury history, play crucial roles in the development and prevention of MSDs. Structured warm-up routines reduce injury risk by enhancing blood flow, increasing muscle elasticity, and improving flexibility, thereby promoting safer performance [16,17]. Managing training intensity is essential, as low-intensity aerobic activity prior to exercise increases body and muscle temperature, promotes changes in active stiffness, and enhances resistance to muscular injuries [17,18]. The history of previous injuries further increases susceptibility to reinjury by altering neuromuscular control and joint stability, which negatively impacts an athlete’s biomechanics [16]. Preventive strategies, such as dynamic stretching, can address these risks effectively [19]. A well-designed flexibility routine primes the body for the strength and power demands of athletic activity and has been shown to reduce injury rates across various sports [19]

Despite these insights, a critical gap remains regarding how these modifiable factors operate specifically among female university athletes, particularly in under-researched contexts such as Saudi Arabia. Given the rapid growth of women’s participation in organized sports nationally, context-specific data are needed to inform prevention and service planning in university athletics. In summary, while sports participation confers clear benefits, the growing recognition and reporting of MSDs in female university athletes highlight the need for targeted, context-specific prevention. Although knowledge of sports-related injury causes has advanced, few studies integrate physiological, biomechanical, and behavioral factors into holistic prevention programs tailored to young adult female university athletes [20,21].

Accordingly, this study (i) estimates the prevalence of MSDs among female university student athletes, (ii) identifies the most commonly affected regions, and (iii) examines associations between MSD occurrence and sport type, adherence to warm-up, weekly training frequency, and prior injury.

## 2. Methodology

### 2.1. Study Design

This study used a descriptive, cross-sectional design to investigate the prevalence, common types, and associated risk factors of musculoskeletal disorders (MSDs) among female university student athletes. The data collection period took four months, from January to April 2025. Ethical approval for this study was obtained from the Institutional Review Board (IRB) of Princess Nourah bint Abdulrahman University (Approval No. 24-0957). Prior to participation, all participants were provided with detailed information about the study’s purpose, procedures, and data confidentiality. Digital informed consent was obtained from each participant electronically, and they were assured that their participation was voluntary and that they could withdraw from the study at any stage without any repercussions.

### 2.2. Participants

The inclusion criteria for this study comprised female university students aged 18 to 25 years, actively enrolled in undergraduate or postgraduate programs at Saudi universities, and regularly participating in organized sports activities or official university sports teams. “Regularly participating” was operationally defined as engaging in organized training for at least three or more sessions per week or a minimum of 180 min per week during the preceding six months. Participants were required to engage in such activities consistently for a minimum of six months before enrollment.

Exclusion criteria included students who did not participate in organized athletic activity, individuals with pre-existing musculoskeletal disorders or injuries unrelated to sports participation, and those diagnosed with chronic medical conditions such as rheumatoid arthritis or osteoarthritis. Pregnant students were also excluded due to the potential influence of pregnancy-related physiological changes on musculoskeletal outcomes.

Sample characteristics including mean age, height, and body mass were recorded and summarized using descriptive statistics to provide a comprehensive profile of the study cohort.

### 2.3. Sample Size Calculation

The total population of university students in Saudi Arabia was reported as 1,297,426 by the Ministry of Education in 2023. Although the exact number of female university athletes remains unavailable, recent reports have noted a steady rise in female participation in sports activities, attributed to national initiatives such as Saudi Vision 2030 [22]. To determine the minimum required sample size, OpenEpi version 3 was utilized. A 95% confidence level and a 5% margin of error were applied. Given the lack of precise prevalence data for this target group, an expected prevalence of 50% was assumed to ensure maximum variability. Based on these parameters, the required minimum sample size was 385 participants. To accommodate potential attrition, including non-response and incomplete questionnaires, the target sample size was increased to 400 participants. This ensures sufficient statistical power to detect significant associations between musculoskeletal disorders and variables such as type of sport, warm-up adherence, training frequency, and injury history.

### 2.4. Sampling Strategy

A probability-based stratified sampling method was employed to ensure balanced representation across various sports disciplines, including team sports (e.g., volleyball, basketball, handball), individual sports (e.g., swimming, track, tennis), strength-training activities, and high-impact sports (e.g., CrossFit, gymnastics, martial arts). Recruitment was conducted across multiple Saudi universities, both public and private, crossing different geographic regions to capture variability in sport types, training environments, and athlete characteristics.

Participants were selected within each stratum (i.e., sport type) using simple random sampling to reduce selection bias and enhance generalizability. This approach ensured that the final sample reflected the diversity of female athletic participation within the university population.

### 2.5. Data Collection Tool

Data was collected using a structured, self-administered questionnaire composed of four sections. The first section included the informed consent statement, ensuring participants understood the study purpose, confidentiality terms, and their right to withdraw at any time.

The second section captured demographic information such as age, height, weight, marital status, educational level, specialization, and sport type.

The third section utilized the Arabic version of the standardized Nordic Musculoskeletal Questionnaire (NMQ) [23], which is a widely used and validated tool for assessing musculoskeletal symptoms across nine anatomical regions: neck, shoulders, elbows, wrists/hands, upper back, lower back, hips/thighs, knees, and ankles/feet. Participants who reported no symptoms in a specific region were automatically directed to skip subsequent questions about that region, thereby streamlining the response process.

Musculoskeletal disorders (MSDs) were defined as any experience of ache, pain, discomfort, or numbness involving soft tissues (muscles, tendons, and ligaments), bones, or joints. Respondents were asked to indicate the presence of symptoms across four timeframes: lifetime, past 12 months, past month, and current day. The NMQ has demonstrated high reliability and validity across populations [24]. The full version of the adapted Arabic questionnaire is available in Al Amer & Alharbi (2023) [25].

The final section assessed potential risk factors for MSDs using a combination of open-ended, close-ended, and multiple-response questions. Topics included the type of sport (e.g., team, individual, or high-impact sports), and warm-up practices (frequency and type). Participants were also asked about previous sports-related injuries, including injury type, severity, and recovery duration. The section concluded with a self-assessment of perceived risk for developing MSDs, rated on a five-point Likert scale from “very low” to “very high”.

### 2.6. Validation of the Tool

To ensure the reliability and contextual relevance of the questionnaire, a pilot study was conducted with 20 female university athlete’s representative of the target population. Participant feedback was used to refine the structure, language clarity, and cultural appropriateness of the survey items. The pilot phase also confirmed the usability of the Arabic version of the NMQ within the study population.

### 2.7. Procedure

Data for this study were collected using an online questionnaire developed via Google Forms to ensure accessibility and facilitate wide distribution. The survey was designed to take approximately 10 to 15 min to complete. The questionnaire link was disseminated through multiple channels, including official university networks, student email lists, social media platforms, and university-affiliated sports clubs. This multi-channel recruitment strategy aimed to reach a diverse and representative sample of female university student athletes across Saudi Arabia. Participants were first directed to a consent page and then asked to complete a screening question to determine eligibility. This binary question confirmed whether respondents met the inclusion criteria. Only those who fulfilled the criteria were allowed to proceed; others were automatically redirected to a message thanking them for their interest. Eligible participants then completed the second section, which collected demographic data and required responses to all items, regardless of perceived relevance. In the third section, which included the Arabic version of the Nordic Musculoskeletal Questionnaire (NMQ), participants were instructed to respond only to questions relevant to the specific body regions where they experienced symptoms. This conditional structure was used to minimize response burden and improve data accuracy. The final section gathered data on modifiable risk factors associated with the development of musculoskeletal disorders, including sport type, warm-up practices, training intensity, and previous injury history.

The questionnaire comprised a total of 124 items, with automated logic functions embedded to tailor the flow of questions based on participant responses. Upon submission, all responses were securely stored, anonymized, and exported for statistical analysis. Data management procedures ensured confidentiality and compliance with ethical standards throughout the research process.

### 2.8. Data Analysis

Data was analyzed using IBM SPSS Statistics version 28 (IBM Corp., Armonk, NY, USA). Descriptive statistics, means, medians, standard deviations, and proportions, were calculated to summarize participants’ demographic characteristics, sports participation patterns, warm-up practices, and the prevalence and impact of MSDs. Categorical variables were expressed as frequencies and percentages, while continuous variables such as age at symptom onset were reported as means and standard deviations.

Normality of continuous variables (e.g., age, training duration) was examined using the Shapiro–Wilk test. As the distributions did not significantly deviate from normality (*p* > 0.05), parametric summaries (mean ± SD) were considered appropriate. In instances of minor skewness, mean and SD were retained for consistency and ease of interpretation, following established recommendations for large-sample descriptive analyses [26,27].

Chi-square tests of independence were used to examine associations between MSD prevalence and sport-related variables, including sport type, warm-up frequency, weekly training frequency, and prior injury history. Variables showing *p*-values ≤ 0.10 in the bivariate analysis were further assessed using univariate logistic regression to estimate odds ratios (ORs) and 95% confidence intervals (CIs).

Variables with *p*-values ≤ 0.10 in the bivariate analysis were retained for inclusion in the univariate logistic regression model to avoid the premature exclusion of potentially meaningful predictors. The choice of an α-level of 0.10 is consistent with established methodological recommendations for exploratory models, where a slightly relaxed threshold helps ensure that relevant variables are carried forward to multivariate analysis [28,29]. This approach is widely used in epidemiological studies to balance statistical rigor with model sensitivity, particularly when assessing multifactorial conditions such as musculoskeletal disorders.

Significant predictors from the univariate models were included in a multivariate logistic regression model to identify independent associations with MSDs while adjusting for potential confounders. A *p*-value of <0.05 was considered statistically significant for all inferential tests.

All statistical procedures adhered to recommended reporting guidelines for observational cross-sectional studies, and findings were presented in alignment with STROBE criteria [30].

## 3. Results

### 3.1. Sample Characteristics

**Figure 1:**
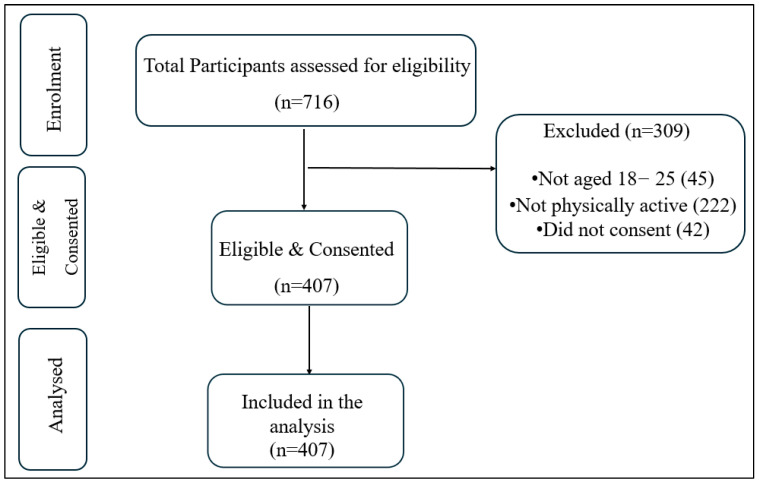
Flowchart of participants’ recruitment.

A total of 716 responses were collected (Figure 1). After applying the inclusion criteria of being between 18 and 25 years of age and regularly participating in physical activity for at least six months, 309 participants were excluded. Specifically, 222 did not regularly participate in physical activity for at least six months, 45 were not aged between 18 and 25 years, and 42 did not consent. The final sample consisted of 407 eligible female university student athletes.

The majority of participants included in this study were between 20 and 23 years old (69.8%), single (96.8%), and enrolled in humanities colleges (52.1%). Most were in their third or fourth academic year. Approximately 70.3% of participants reported participating in organized or collegiate-level sports, while 12.0% reported a history of musculoskeletal issues unrelated to sports participation (Table 1).

### 3.2. Prevalence and Types of Musculoskeletal Disorders

**Figure 2:**
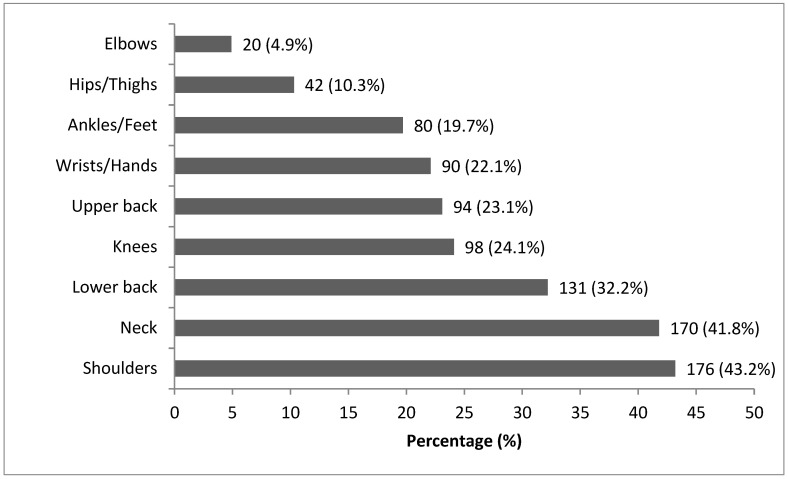
Prevalence of Musculoskeletal Disorders by region (*n* = 407).

Of the 407 participants, 268 (65.8%) reported experiencing at least one musculoskeletal disorder (MSD) within the past 12 months (Table 1). The most frequently affected regions were the shoulders (43.2%), neck (41.8%), and lower back (32.2%), followed by the knees, upper back, wrists/hands, ankles/feet, hips/thighs, and elbows, as presented in (Figure 2).

### 3.3. Prevalence and Impact of MSDs by Body Region

Data from the Nordic Musculoskeletal Questionnaire is summarized in Table 2. The average age of symptom onset ranged from 18.3 to 19.2 years across body regions. Pain experienced within the last 12 months was most commonly reported in the neck (30.1%), shoulders (29.4%), and lower back (23.0%). In terms of more recent symptoms, 24.5% of participants reported neck pain within the last four weeks, while 22.8% reported shoulder pain and 19.8% reported lower back pain.

Current discomfort on the day of survey completion was reported by 13.0% for neck, 12.5% for shoulders, and 10.3% for lower back. Several participants reported being prevented from performing daily activities due to these symptoms, including 13.5% of those with shoulder pain and 12.5% with neck pain. Changes in job duties or daily responsibilities due to MSDs were reported by 19.8% of those with shoulder symptoms, 17.2% with neck pain, and 17.6% with lower back pain. Healthcare utilization was evident, as 15.1% of participants with upper back or wrist/hand pain reported visiting a professional. Medication use was also notable, with 10.7% using medication for shoulder pain and 9.8% for neck pain. Sick leave due to musculoskeletal complaints was most frequently reported among those with ankle/foot issues (7.1%) and neck pain (5.1%). Although rare, hospitalization due to MSDs was reported in multiple body regions, with rates ranging from 0.5% to 1.9%.

### 3.4. Sports Participation and Warm-Up Practices

As detailed in Table 3, the most commonly practiced sport type was team sports (35.1%), followed by individual sports (33.1%) and strength training (24.5%). About one-third of participants reported exercising three days per week, with 26.0% exercising four days and 24.8% exercising five days or more. The majority of participants (52.6%) exercised for more than 60 min per session.

Warm-up practices were reported by 62.9% of the participants. Stretching was the most common form of warm-up (43.6%), followed by cardio (38.0%) and light exercise (10.9%). Despite this, only 22.6% of participants reported warming up more than five times per week. Most participants warmed up for 5–10 min (49.8%), while 36.6% reported a warm-up duration of less than five minutes.

### 3.5. Chi-Square Tests of Association

The chi-square analysis (Table 4) revealed a significant association between MSD prevalence and previous injury history (χ^2^ (1) = 15.90, *p* < 0.001), Whereas type of sport, warm-up frequency, and weekly exercise frequency were not significantly related to MSD occurrence. This finding indicates that participants who had sustained a prior injury were more likely to report current MSDs.

As shown in Table 5, logistic regression analysis identified previous injury history and weekly training as a significant predictor of MSDs occurrence. Participants with a history of injury were more than twice as likely to report current MSDs, and those with higher weekly training frequency also showed increased odds. Participation in team sports demonstrated a borderline association, whereas warm-up frequency, session duration, and strength training were not significantly related to MSD prevalence.

## 4. Discussion

This study revealed a high prevalence of musculoskeletal disorders (MSDs) among female university student athletes (65.8%), particularly affecting the shoulders, neck, and lower back. These findings indicate that MSDs represent a substantial concern for young female athletes in Saudi Arabia and underscore the need for sport-specific preventive and rehabilitative strategies. The observed prevalence aligns closely with prior university-based studies such as Alshagga et al. (2013), who reported MSD rates of 64.8% among students, confirming the global burden of musculoskeletal complaints in this age group [31].

The present study expands on existing evidence by identifying previous injury history and weekly training frequency as the strongest predictors of MSD occurrence. Athletes with a prior injury were more than twice as likely to experience new or persistent MSDs, reinforcing the injury-predisposition hypothesis, in which earlier trauma compromises tissue integrity and neuromuscular control, predisposing athletes to recurrent problems [32]. Likewise, higher weekly training frequency was significantly associated with MSD risk, consistent with the workload–injury causation model, which links excessive or rapidly increased training volumes to overuse injuries [33,34]. Together, these findings emphasize the need for structured load-monitoring and individualized recovery protocols to maintain training adaptation without exceeding musculoskeletal tolerance [35].

The anatomical distribution of MSDs in this study mirrors established patterns reported in the literature. The predominance of shoulder, neck, and lower-back symptoms may reflect the biomechanical and postural demands of common university-level sports. For example, repetitive overhead movements in volleyball or throwing sports increase shoulder strain, predisposing to rotator cuff overload and impingement syndromes [36]; limited cervical muscle endurance and forward head posture, commonly observed in female athletes, may contribute to neck discomfort [37]; and axial loading with trunk rotation typical of court or field sports can exacerbate lower-back pain [38,39]. Although our study did not assess these specific pathologies directly, these mechanisms provide plausible explanations for the anatomical trends observed.

Interestingly, sport type was not significantly associated with MSD prevalence in the current sample, though participation in team sports approached significance. This finding may reflect the higher physical intensity, frequent contact, and coordination demands inherent in team-based activities, partially supporting prior evidence that contact or high-impact sports elevate musculoskeletal risk [40]. The absence of a significant difference may, however, result from heterogeneity within sport categories or unequal sample sizes between groups.

Warm-up practices were not found to be significantly protective, which diverges from evidence demonstrating that structured dynamic warm-ups, such as FIFA 11+, effectively reduce injury risk [41]. This discrepancy likely stems from the short duration (≤10 min) and predominantly static stretching routines reported by participants, which may not reach the physiological thresholds required for neuromuscular activation and tissue preparation [41,42].

Beyond prevalence, MSDs in this population carried functional consequences. Participants frequently reported limitations in daily activity, reliance on medication, and healthcare visits, particularly for neck and shoulder pain. These functional outcomes corroborate Bajwa (2023), who associated chronic MSD symptoms with reduced academic engagement and increased absenteeism among university students [42]. From a public-health perspective, these findings support integrating early injury screening, load-management education, and structured warm-up programs within university athletic systems. Interdisciplinary collaboration among physical therapists, sports physicians, and coaches is essential to minimize MSD risk and promote long-term musculoskeletal health in female university athletes. It is also important to acknowledge the limitations of this study, the cross-sectional design prevents causal inference, and the reliance on self-reported data may introduce recall or reporting bias. Although a large number of students were initially approached, the proportion who completed the questionnaire was comparatively lower, which may introduce participation bias if those who responded differ systematically from those who did not. Additionally, while the sample was diverse in terms of sport type and college affiliation, all participants were female university students from a single national context, limiting generalizability. Nonetheless, the robust sample size (*n* = 407), use of validated instruments (Nordic Musculoskeletal Questionnaire), and multivariate modeling strengthen the internal validity of the findings. Future studies should use longitudinal designs to establish causal relationships between training load, injury history, and MSD development. Incorporating objective assessments (e.g., motion analysis or load monitoring) would enhance data accuracy. Research should also examine the effectiveness of structured warm-up programs and explore gender-specific risk factors. Expanding the sample to include diverse populations and qualitative insights could provide a more comprehensive understanding of MSD prevention in student athletes.

## 5. Conclusions

This study confirms a high prevalence of MSDs among female university student athletes, with the shoulders, neck, and lower back most commonly affected. History of previous injury and higher weekly training frequency were identified as significant independent predictors of MSD occurrence, while team sport participation demonstrated a borderline association that warrants further investigation. Although warm-up frequency and sport type were not statistically significant predictors, these variables merit further exploration using more detailed behavioral and biomechanical assessment tools. These findings underscore the importance of implementing targeted prevention strategies, emphasizing rehabilitation continuity, particularly for athletes with prior injuries, and training load management for those with higher weekly training demands. Such approaches can help mitigate the burden of MSDs and promote sustainable athletic performance in this under-researched population.

## Figures and Tables

**Table 1:** Demographic characteristics of study participants (*n* = 407).

Cat.	Subcat.	*n*	%
**Age**	16–19	90	22.1
	20–23	284	69.8
	>23	33	8.1
**Marital Status**	Single	394	96.8
	Married	10	2.4
	Divorced	2	0.5
	Widowed	1	0.3
**College**	Health Science	103	25.3
	Scientific	63	15.5
	Literacy	29	7.1
	Humanities	212	52.1
**Academic Year**	1	71	17.4
	2	76	18.7
	3	106	26
	4	144	35.4
	5	7	1.7
	6	3	0.7
**Regular Exercise**	Yes	286	70.3
	No	121	29.7
**History of Non-Sport MSD**	Yes	49	12
	No	358	88
**MSD (Sport-related)**	Yes	268	65.8
	No	139	34.2

Note: Cat. = Category; Subcat. = Subcategory; n = Frequency; % = Percentage.

**Table 2:** Prevalence and Impact of Musculoskeletal disorders by Body Region (*n* = 407).

Body Region	Age of Initial Onset *n* (sd)	Discomfort Today *n* (%)	Hospitalization *n* (%)	Change in Job or Duties *n* (%)	Problems in Last 4 Weeks *n* (%)	Pain During Last 12 Months *n* (%)	Prevented from Usual Activity *n* (%)	Seen Professional Help *n* (%)	Taken Medication *n* (%)	Taken Sick Leave *n* (%)
**Neck**	18.3 (2.4)	53 (13.0)	5 (1.2)	70 (17.2)	100 (24.5)	123 (30.1)	51 (12.5)	29 (7.1)	40 (9.8)	21 (5.1)
**Shoulder**	18.5 (3.0)	51 (12.5)	8 (1.9)	81 (19.8)	93 (22.8)	120 (29.4)	55 (13.5)	40 (9.8)	44 (10.7)	20 (4.9)
**Upper back**	18.6 (3.1)	25 (6.1)	4 (1.0)	38 (9.3)	43 (10.5)	57 (14.0)	18 (4.4)	21 (15.1)	17 (4.2)	12 (2.9)
**Elbows**	18.9 (3.2)	7 (1.7)	6 (1.5)	12 (2.9)	10 (2.4)	15 (3.6)	10 (2.4)	5 (1.2)	5 (1.2)	3 (0.7)
**Wrists hands**	19.1 (2.1)	26 (6.4)	7 (1.7)	55 (13.4)	45 (11.0)	65 (15.9)	37 (9.1)	21 (15.1)	18 (4.4)	9 (2.2)
**Lower back**	18.7 (3.2)	42 (10.3)	5 (1.2)	72 (17.6)	81 (19.8)	94 (23.0)	46 (11.3)	33 (8.1)	30 (7.3)	16 (3.9)
**Hips Thighs**	19.2 (2.6)	11 (2.7)	2 (0.5)	27 (6.6)	28 (6.8)	35 (8.5)	25 (6.1)	20 (4.9)	18 (4.4)	9 (2.2)
**Knees**	18.4 (2.9)	28 (6.8)	8 (1.9)	57 (13.9)	54 (13.2)	74 (18.1)	40 (9.8)	28 (6.8)	25 (6.1)	14 (3.4)
**Ankle feet**	18.5 (2.8)	33 (8.1)	8 (1.9)	58 (14.2)	52 (12.7)	62 (15.2)	48 (11.7)	36 (8.8)	38 (9.3)	29 (7.1)

**Table 3:** Sports Participation and Warm-Up Characteristics (*n* = 407).

Variable	Category	Frequency (*n*)	Percentage (%)
**Sport Type**	Team Sports	143	35
	Individual Sports	135	33
	Strength Training	100	25
	Other	29	7
**Weekly Training Frequency**	1–2 days	65	16
	3 days	135	33
	4 days	106	26
	≥5 days	101	25
**Session Duration**	<30 min	44	10.8
	30–60 min	149	36.6
	>60 min	214	52.6
**Warm-Up Participation**	Yes	256	62.9
	No	151	37.1
**Warm-Up Type**	Stretching	112	43.6
	Light Cardio	97	38
	Light Strength Exercises	28	10.9
	Other	19	7.4
**Warm-Up Frequency per Week**	<1 time	85	20.9
	1–2 times	104	25.6
	3–4 times	92	22.6
	≥5 times	92	22.6
**Warm-Up Duration**	<5 min	149	36.6
	5–10 min	203	49.8
	>10 min	55	13.5

**Table 4:** Association Between Musculoskeletal Disorder Prevalence and Sport-Related Variables Based on Chi-Square Test (*n* = 407).

Variable	Chi-Square (χ^2^)	df	*p*-Value
**Type of Sport**	6.44	3	0.09
**Warm-up Frequency**	0.33	2	0.85
**Weekly Training Frequency**	7.45	4	0.11
**Previous Injury History**	15.90	1	<0.001

**Table 5:** Logistic Regression Analysis of Factors Associated with MSDs (*n* = 407).

Predictor	Univariate OR (95% CI)	*p*-Value	Multivariate OR (95% CI)	*p*-Value
**Previous injury history**	2.67 (1.63–4.37)	<0.001	2.44 (1.47–4.06)	0.001
**Weekly training frequency**	1.27 (1.06–1.52)	0.011	1.23 (1.02–1.49)	0.034
**Team sport participation**	1.82 (1.10–2.99)	0.019	1.59 (0.95–2.67)	0.079
**Strength training**	1.05 (0.65–1.70)	0.84	1.02 (0.62–1.67)	0.93
**Warm-up frequency**	0.96 (0.77–1.19)	0.72	0.94 (0.74–1.18)	0.59
**Session duration**	1.01 (0.83–1.23)	0.91	0.99 (0.81–1.21)	0.88

## Data Availability

The datasets used and/or analyzed during the current study are available from the corresponding author on reasonable request.
